# Comparison of conventional echocardiographic parameters of RV systolic function with cardiac magnetic resonance imaging

**DOI:** 10.1186/1532-429X-17-S1-M10

**Published:** 2015-02-03

**Authors:** Xiao Zhou, Shizhen Liu, Zhen Qian, James C Lee, Robi Goswami, Ashish Kabra, Raul R Blanco, Jyoti Sharma, Mani Vannan, Sarah Rinehart, Venkateshwar Polsani

**Affiliations:** 1 Cardiac CT/MR Imaging, Piedmont Heart Institute, Atlanta, GA, USA; 2 Cardiology, University Of Washington, Seatle, WA, USA

## Background

Cardiac magnetic resonance (CMR) imaging is the reference standard to assess right ventricular (RV) volumes and ejection fraction. However, 2-D echocardiography is commonly used for routine assessment of the RV and a number of quantitative measures have been recommended to evaluate systolic function.^1^ Measurement of right ventricular ejection fraction (RVEF), which is a key predictor of outcomes in a range of right heart diseases, is not recommended because of the limitations of 2-D imaging of the RV. Instead Fractional Area Change (FAC %)by 2-D Echocardiography and tricuspid annular plane systolic excursion (TAPSE) are recommended as surrogate measures of RV global systolic function. The aim of our study is to compare the conventional parameters of RV systolic function currently used by 2-D echocardiography with RVEF and stroke volume (SV) measured by CMR.

## Methods

A total of 125 consecutive patients (from November 2013 to July 2014) who consented for the CMR registry at Piedmont Heart Institute were reviewed for this study. 72 patients with adequate RV function assessment by 2-D echocardiography and CMR were included. 2-D echocardiography RV FAC (%), and TAPSE (mm) measurements were compared with CMR RVEF (%) and SV (ml). The comparison was made using linear correlation for the echo variables with CMR variables. FAC was then compared with CMR RVEF using inter-rater agreement (kappa).

## Results

Table 1 shows the baseline demographic characteristics of the patients. Table 2 shows the RV function by 2-D Echocardiography and CMR. 85% of patients had normal RVEF by CMR. There was poor correlation between RV function measurements by 2-D echocardiography with RVEF and SV as calculated by CMR. TAPSE did not correlate with either RVEF or SV by CMR. FAC correlated the best with RV EF (R =0.43, p= 0.0003) by CMR, but not with SV. When FAC was compared with RV EF, using inter-rater agreement (kappa) statistic, there was fair agreement (kappa 0.234). CMR RVEF reclassified RV function as assessed by FAC in 20% of patients. 10% (n=7) of patients were reclassified as normal and another 10% (n=7) were reclassified as abnormal.

**Table 1:** Demographic characteristics of the study population

	n	Mean ± SD
Age (y)	72	55.47±16.22

Male Gender	37 (51.4%)	

Height (cm)	72	171.68±11.03

Weight (kg)	72	77.88±5.00

Body Surface Area (m2)	72	1.92±0.15

Body Mass Index (BMI)	72	26.54±1.69

Race		

White	56 (77%)	

African American	15 (21%)	

History of Hypertension	41 (56.9%)	

History of Hyperlipidemia	24 (33%)	

History of Diabetes	4 (5.6%)	

No Smoking History	46 (63.9%)	

Family History of CAD	31 (43.1%)	

LV systolic Failure	15 (20.8%)	

**Table 2:** RV function evaluation from Echo and CMR

	n	Mean ± SD	r (compared to CMR-EF)	r (compare to CMR_SV)	Kappa(compared to CMR-EF)
Echo					

FAC (%)	65	42.86 ± 9.15	0.43 (p=0.003)	0.10 (p=0.39)	0.234

TAPSE (mm)	47	19.56 ± 5.44	0.12 (p=0.40)	0.16 (p=0.40)	

S' (cm/s)	45	12.44 ± 3.17			

RA area (cm2)	71	15.72 ± 4.19	0.50 (p=0.0002)#		

CMR					

EF (%)	72	53.61 ± 11.56			

SV (ml)	72	85.74 ± 25.79			

RA area (cm2)	52	23.52 ± 6.42			

# compared to CMR_RA area (n=52)

## Conclusions

The current 2-D echocardiographic parameters of RV systolic function assessment correlate poorly with CMR measured RVEF and SV.CMR should be utilized more often to measure RVEF and volumes to complement routine 2-D echocardiography measurements for comprehensive and accurate evaluation of RV systolic function.

## Funding

N/A.

